# Risk of epilepsy in people with adult-onset hydrocephalus: insights from the UK Biobank

**DOI:** 10.1007/s10072-026-09227-6

**Published:** 2026-07-16

**Authors:** Jolanda Buonocore, Francesco Fortunato, Enrico Fratto, Ilaria Sammarra, Antonio Gambardella, Aldo Quattrone, Andrea Quattrone

**Affiliations:** 1 Institute of Neurology, Department of Medical and Surgical Sciences, University ″Magna Graecia″, Catanzaro, Italy; 2 Neuroscience Research Centre, University ″Magna Graecia″, Catanzaro, Italy

**Keywords:** Adult-onset hydrocephalus, Epilepsy, Seizures, Neurodegeneration, UK Biobank

## Abstract

**Aim:**

We aim to determine whether individuals with adult-onset hydrocephalus have an increased risk of incident epilepsy over time.

**Methods:**

We analysed data from the UK Biobank cohort. Diagnoses of “hydrocephalus” and “epilepsy” were identified through ICD-10-coded health records, excluding congenital and secondary cases. The association between these two diseases was investigated using logistic regression (adjusted for age and sex); subsequently, we performed a sampled cohort study to evaluate the hazard ratio (HR) for incident epilepsy in people with adult-onset hydrocephalus using Cox proportional hazards models with a dedicated sensitivity analysis including different sets of covariates such as demographic, lifestyle, vascular, and genetic factors.

**Results:**

Our cohort included 483,790 controls, 5,028 individuals with epilepsy, and 320 with adult-onset hydrocephalus. Hydrocephalus and epilepsy were strongly associated (OR: 9.6, 95% CI: 6.4–13.8, *p* < 0.001). Adjusted Cox models demonstrated a markedly increased risk of incident epilepsy in people with adult-onset hydrocephalus, with HR values ranging from 14.62 (95% CI: 7.91–27.00, *p* < 0.001) to 23.80 (95% CI: 12.87–44.03, *p* < 0.001) across models, after adjusting for multiple covariates. Results were also consistent after excluding people with comorbid neurodegenerative dementias such as Alzheimer’s disease.

**Conclusions:**

We demonstrated a markedly increased risk of epilepsy in adult-onset hydrocephalus, independent of vascular and neurodegenerative comorbidities and not attributable to shunt-related complications. These findings underscore the importance of increased diagnostic vigilance, as seizures may be under-recognized in this population, and support further research to clarify mechanisms and optimize management strategies.

**Supplementary Information:**

The online version contains supplementary material available at https://doi.org/10.1007/s10072-026-09227-6.

## Introduction

Idiopathic normal pressure hydrocephalus (iNPH) is an adult-onset disease, classically characterized by the triad of gait impairment, cognitive decline, and urinary disturbances [1]. Population-based studies have reported a clinical diagnosis of iNPH prevalence of approximately 1–5% among individuals aged 65 years or older and an incidence of nearly 5–7 per 100,000 person/years, with higher rates observed in older age groups, especially those over 80 years, where prevalence may approach 6–8% [2–5]. These values may also be understated, as more recent MRI-based reports have revealed higher frequencies when radiological or subclinical forms are included [6, 7]. INPH has recently gained a growing interest in the neurology community due to the clinical and radiological overlap with neurodegenerative parkinsonism and dementias, with several studies investigating their clinical spectrum, as well as radiological biomarkers [8–10].

Epilepsy is also a highly impactful condition in older adults, with incidence rising steadily after the age of 50 and peaking in individuals over 75 years. It is the fifth and seventh leading cause of disability-adjusted life years (DALYs) in those aged 60–79 years and in those over 80 years, respectively [11]. Late-onset epilepsy (LOE) is usually symptomatic, most commonly resulting from structural brain damage [12]. Cerebrovascular disease accounts for a substantial proportion of these cases [12–14], but a proportion of LOE cases remain classified as of unknown aetiology [14]. Growing interest has therefore focused on the association between LOE and neurodegenerative conditions, particularly Alzheimer’s disease (AD), with increasing evidence suggesting that epilepsy may represent an early clinical manifestation of an underlying neurodegenerative process [15–18].

Epilepsy has previously been reported in patients with hydrocephalus, but most data are from paediatric populations or from shunt-related complications [19, 20]. Consequently, little is known about the co-occurrence of epilepsy and idiopathic adult-onset hydrocephalus. Clarifying whether iNPH is associated with an increased risk of epilepsy has important clinical implications, as it could contribute to earlier diagnosis and support the timely initiation of anti-seizure therapy. Moreover, understanding the relationship between iNPH and epilepsy may be crucial when considering shunt surgery: while surgical intervention could potentially exacerbate seizure susceptibility in some people, it may also influence epileptic activity when ventriculomegaly is the potential underlying cause.

Building on this rationale, we used data from the UK Biobank, a large prospective population-based cohort with linked hospital and primary care records [21], to investigate the relationship between adult-onset hydrocephalus and epilepsy and to determine whether individuals with hydrocephalus have an increased risk of incident epilepsy over time.

## Methods

### Cohort selection

This study used data from the UK Biobank, a large population-based cohort of approximately 500,000 participants with an age at recruitment of 40–69 years, enrolled between 2006 and 2010 from 22 assessment centres across the United Kingdom [21].

The inclusion and exclusion criteria of the present study were defined after a dedicated consensus agreement between two neurologists with expertise in epilepsy (i.e., FF and IS) and two with expertise in hydrocephalus and neurodegenerative conditions (i.e., AQ and JB). Only when the entire study team agreed on a proposed criterion was it adopted for use in the present study.

**Fig. 1:**
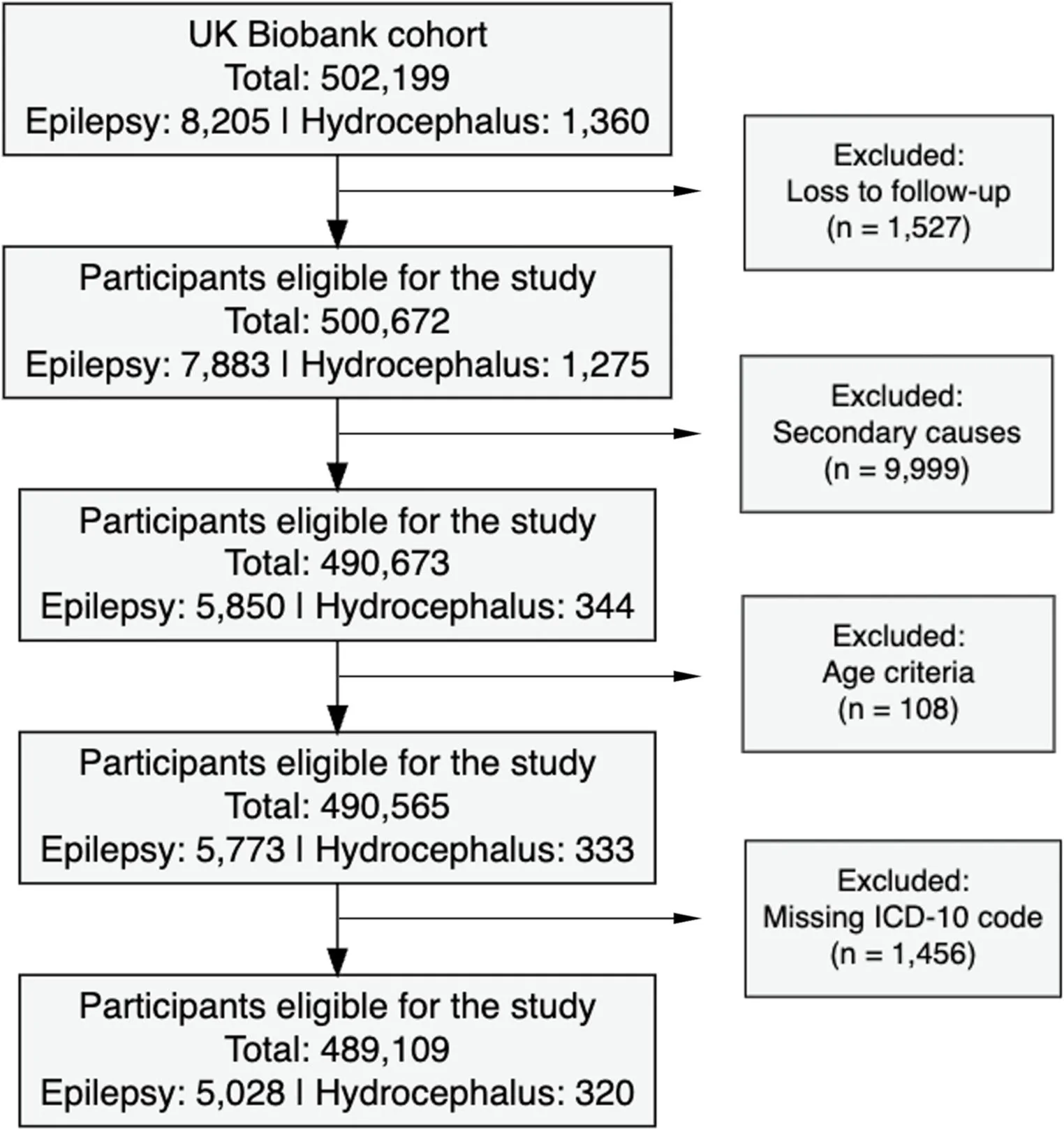
Flowchart of participant selection from the UK Biobank cohort. Starting from 502,199 participants, individuals were sequentially excluded due to loss to follow-up, secondary causes of hydrocephalus or epilepsy, age criteria, and missing ICD-10 diagnostic codes. Our definite cohort included 489,109 participants, of whom 5,028 had epilepsy and 320 had hydrocephalus

The inclusion criteria were the presence of a diagnosis of hydrocephalus (International Classification of Diseases, 10th Revision [ICD-10] code: G91) and/or epilepsy (ICD-10 code: G40) as identified through linked hospital inpatient, primary care and death registry records, excluding self-reported diagnosis records. Individuals with no hydrocephalus or epilepsy diagnosis were included as controls. Exclusion criteria were: (a) loss to follow-up; (b) secondary causes of hydrocephalus or epilepsy, including structural brain lesions (e.g. tumors, intracranial haemorrhage, congenital malformations), systemic diseases, and non-idiopathic forms of hydrocephalus. For conditions with a definable acute onset, such as meningitis, encephalitis, or cerebrovascular disease, exclusion was restricted to cases recorded prior to or within 30 days of the hydrocephalus or epilepsy diagnosis date; for all other secondary causes whose diagnosis dates were not available, exclusion was applied regardless of timing (Supplementary Table 1); (c) disease-specific age exclusions: epilepsy diagnosed before the age of 2 years, to reduce the likelihood of cases with a monogenic epilepsy aetiology [22, 23]; hydrocephalus diagnosed before the age of 40 years, as earlier-onset cases are more likely to reflect secondary or congenital forms rather than late-onset idiopathic hydrocephalus; (d) conflicting data about diagnosis of interest (i.e. presence of epilepsy or hydrocephalus diagnosis date but no corresponding ICD-10 code). The full exclusion process is summarized in ( Fig. 1), and the complete list of ICD-10 codes excluded is provided in Supplementary Table 1. The resulting cohort was used to estimate the overall association between hydrocephalus and epilepsy. 

We used “adult-onset hydrocephalus” through the manuscript to reflect the nature of the data extracted from the population-based UK Biobank initiative, but we are confident our findings can be interpreted in the context of iNPH which is the typical form of hydrocephalus with onset above 40 years in the absence of secondary causes (all consistent with our inclusion/exclusion procedures).

### Sampled cohort study

We performed a sampled cohort study to investigate whether individuals with adult-onset hydrocephalus were at higher risk for incident epilepsy compared with matched controls without hydrocephalus. Individuals with hydrocephalus were considered at risk from the date their diagnosis was first reported, while controls were identified among participants without hydrocephalus and sampled in a 1:100 ratio. Five independent matched datasets were generated using different random seeds to account for the variability introduced by random sampling. Each control was assigned a pseudo-index date corresponding to a surrogate age at diagnosis, sampled from the distribution of ages at hydrocephalus diagnosis observed among the cases. The rationale for this approach was to ensure comparability in the definition of time at risk between cases and controls [24]. This procedure aligned the starting point of follow-up across groups, thereby enabling valid estimation of hazard ratios (HRs) in Cox regression analyses. Further details on the methodology are provided in the Supplementary Materials.

Because of the well-recognized interplay between epilepsy and AD, where seizures may precede or accompany dementia [25, 26], and the well-known diagnostic overlap between idiopathic normal pressure hydrocephalus and AD [27, 28], we performed a sensitivity analysis excluding participants with a diagnosis of AD and other neurodegenerative disorders from both the control and hydrocephalus groups.

### Statistical analysis

Descriptive analyses were conducted to compare participants according to hydrocephalus and epilepsy status. Categorical variables were summarized as counts and percentages, with group differences assessed using Chi-square tests; Cramér’s V was calculated to quantify effect sizes for categorical comparisons. Continuous variables were assessed for normality using the Shapiro–Wilk test or the Kolmogorov–Smirnov test, as appropriate, and were summarized as means and standard deviations and compared between groups using t-test or the Wilcoxon rank-sum test. For continuous variables, Cohen’s d was used to estimate effect sizes, with d ≥ 0.2 interpreted as a small but meaningful effect. All reported p-values were adjusted for multiple testing using the false discovery rate (FDR), with statistical significance set at *p* < 0.05 after correction. Adjusted values are reported as q-values. To investigate the relationship between adult-onset hydrocephalus and epilepsy, we employed two complementary modelling strategies. First, logistic regression was used to estimate the overall association between hydrocephalus and epilepsy, irrespective of the temporal sequence of diagnoses. This model yielded odds ratios (ORs) with 95% confidence intervals (CIs), adjusted for age and sex.

Second, time-to-event analyses were performed using Cox proportional hazard models within sampled cohort structure described above. A series of models was developed progressively, beginning with a baseline model that included only a hydrocephalus diagnosis, followed by sequential adjustment for age and sex, socioeconomic status (Townsend deprivation index, a composite measure based on unemployment, non-home ownership, household overcrowding, and lack of car access, in which higher values indicate greater deprivation) and ethnicity, lifestyle factors (smoking and alcohol consumption), clinical comorbidities (diabetes and hypertension), and genetic variables including APOE ε4 carrier status, polygenic risk score for Alzheimer’s disease, and family history of dementia.

Finally, the fully adjusted model included all covariates simultaneously. Analyses were performed across the five matched datasets to evaluate HR consistency. Please refer to Supplementary Table 2 for the UK Biobank fields used in the analyses and to Supplementary Materials for additional methodological details. Proportional hazards assumptions were assessed for all Cox models using Schoenfeld residuals. All statistical analyses were conducted in R (version 2025.09.0.387). Further details on the Cox models and analyses across the five datasets are provided in the Supplementary Materials.

## Results

### Cohort description

Our study population consisted of 483,790 controls without epilepsy or hydrocephalus, 5,028 individuals with epilepsy, and 320 individuals with hydrocephalus. Demographic and clinical characteristics of these groups are reported in Table 1. Compared with controls, participants with epilepsy were slightly older at baseline and included a marginally lower proportion of females. They also showed greater Townsend index (− 0.59 vs. −1.32, *p* < 0.001) and a higher prevalence of smoking (14.5% vs. 10.4%, *p* < 0.001), lower alcohol consumption (83.3% vs. 91.8%, *p* < 0.001), and more frequent vascular comorbidities, including diabetes (15.5% vs. 9.1%, *p* < 0.001) and hypertension (47.7% vs. 31.8%, *p* < 0.001). Individuals with adult-onset hydrocephalus were older than controls at baseline (60.3 vs. 57.0 years, *p* < 0.001), had higher rates of diabetes (19.1% vs. 9.1%, *p* < 0.001) and hypertension (57.5% vs. 31.8%, *p* < 0.001). No meaningful differences emerged between either epilepsy or hydrocephalus groups and controls with respect to family history of dementia or the polygenic risk score for Alzheimer’s disease (please see Table 1). All these differences were small, and the Townsend Index only showed a significant (> 0.2) effect size, making it unlikely for such group differences to affect the main study results.

**Table 1:** Baseline characteristics of study participants

Data	Controls (*n* = 483,790)	Epilepsy (*n* = 5,028)	Hydrocephalus (*n* = 320)	*p* value (CS vs. Epilepsy)	*p* value (CS vs. Hydrocephalus)
Sex (% Female)	264,528 (54.7)	2,559 (50.9)	165 (51.6)	**< 0.001** ^**a**^	0.29^a^
Age at baseline visit, ys^*^	57.0 (8.1)	57.7 (8.1)	60.3 (6.9)	**< 0.001** ^**c**^	**< 0.001** ^**b**^
Age at hydrocephalus diagnosis, ys^*^	-	64.5 (7.0)	67.8 (9.0)	-	-
Age at epilepsy diagnosis, ys^*^	-	48.3 (22.0)	57.2 (17.7)	-	-
Townsend Deprivation Index^*^	-1.32 (3.1)	-0.59 (3.4)	-1.25 (3.1)	**< 0.001** ^**c**^	0.65^b^
Ethnicity (% White)	456,800 (94.4)	4,781 (95.1)	307 (95.9)	**0.04** ^**a**^	0.29^a^
Current smokers (%)	50,249 (10.4)	727 (14.5)	38 (11.9)	**< 0.001** ^**a**^	0.43^a^
Current alcohol (%)	444,051 (91.8)	4,187 (83.3)	283 (88.4)	**< 0.001** ^**a**^	0.38^a^
Diabetes (%)	44,020 (9.1)	780 (15.5)	61 (19.1)	**< 0.001** ^**a**^	**< 0.001** ^**a**^
Hypertension (%)	153,727 (31.8)	2,396 (47.7)	184 (57.5)	**< 0.001** ^**a**^	**< 0.001** ^**a**^
ApoE-ε4 carrier (%)	106,355 (22.0)	1,172 (22.0)	76 (23.8)	**0.03** ^**a**^	0.49^a^
Family history of AD/dementia (%)	56,163 (11.6)	604 (12.0)	40 (12.5)	0.39^a^	0.68^a^
PRS for AD^*^	0.05 (1.0)	0.09 (1.0)	0.10 (1.0)	0.09^b^	0.65^b^

Participants were categorized into three groups: controls without epilepsy or hydrocephalus (CS), epilepsy (including individuals with epilepsy regardless of hydrocephalus status), and hydrocephalus (including individuals with hydrocephalus regardless of epilepsy status). Values are reported as mean ± standard deviation for continuous variables and as number (percentage) for categorical variables. Descriptions and UK Biobank field identifiers for all variables are provided in Supplementary Table 2. All reported p values were adjusted for multiple testing using the false discovery rate (FDR). All significant associations remained significant after FDR correction, except for ethnicity in the comparison between controls and epilepsy.  Abbreviations: *CS*  controls, *PRS* polygenic risk score, *AD* Alzheimer’s disease. a chi-squared test b Wilcoxon rank-sum test c t-test

We further conducted comparisons across mutually exclusive groups: individuals with epilepsy only, those with hydrocephalus only, and those with both conditions (Table 2). In these analyses, individuals with epilepsy and co-occurring hydrocephalus had a later age at epilepsy onset than those without hydrocephalus (57.2 vs. 48.2 years, *p* = 0.03). Conversely, among participants with hydrocephalus, those with epilepsy had a younger age at hydrocephalus diagnosis compared to those without epilepsy (64.5 vs. 68.1 years, *p* = 0.01) (Table 2). No other demographic, lifestyle, or vascular risk factors differed significantly between subgroups.

**Table 2:** Pairwise comparisons of baseline characteristics between participants with epilepsy, hydrocephalus, and both conditions

Data	Epi only (*n* = 4,999)	Hydro only (*n* = 291)	Hydro + Epi group (*n* = 29)	*p* value (Epi only vs. Hydro + Epi)	*p* value (Hydro only vs. Hydro + Epi)
Sex (% Female)	2,544 (50.9)	150 (51.5)	15 (51.7)	1.0^a^	1.0^a^
Age at baseline visit, ys^*^	57.7 (8.1)	60.3 (6.9)	60.1 (5.9)	0.17^b^	0.62^b^
Age at hydrocephalus diagnosis, ys^*^	-	68.1 (9.1)	64.5 (7.0)	-	**0.01** ^**b**^
Age at epilepsy diagnosis, ys^*^	48.2 (22.0)	-	57.2 (17.7)	**0.03** ^**b**^	-
Townsend Deprivation Index^*^	-0.59 (3.4)	-1.25 (3.1)	-1.28 (3.1)	0.39^b^	0.90^b^
Ethnicity (% White)	4,752 (95.1)	278 (95.5)	29 (100.0)	0.40^a^	0.62^a^
Current smokers (%)	724 (14.5)	35 (12.0)	3 (10.3)	0.79^a^	1.0^a^
Current alcohol (%)	4,165 (83.3)	261 (89.7)	22 (75.9)	0.31^a^	0.06^a^
Diabetes (%)	775 (15.5)	56 (19.2)	5 (17.2)	0.80^a^	0.99^a^
Hypertension (%)	2,379 (47.6)	167 (57.4)	17 (58.6)	0.32^a^	1.0^a^
ApoE-ε4 carrier (%)	1,164 (23.3)	68 (23.4)	8 (27.6)	0.74^a^	0.78^a^
Family history of AD/dementia (%)	601 (12.0)	37 (12.7)	3 (10.3)	1.0^a^	1.0^a^
PRS for AD^*^	0.09 (1.0)	0.08 (1.0)	0.24 (1.2)	0.43^b^	0.40^b^

Participants were categorized into three mutually exclusive groups: epilepsy without hydrocephalus, hydrocephalus without epilepsy and hydrocephalus with epilepsy. Values are reported as mean ± standard deviation for continuous variables and as number (percentage) for categorical variables. Descriptions and UK Biobank field identifiers for all variables are provided in Supplementary Table 2. All reported p-values were adjusted for multiple testing using the false discovery rate (FDR), and no significant p-values remained statistically significant after FDR correction. Abbreviations: *Epi* only epilepsy without hydrocephalus, *Hydro* only hydrocephalus without epilepsy, *Hydro* + *Epi* hydrocephalus with epilepsy, *PRS* polygenic risk score, *AD *Alzheimer’s disease

^a^ chi-squared test

^b^ Wilcoxon rank-sum test

### Association between hydrocephalus and epilepsy

**Fig. 2:**
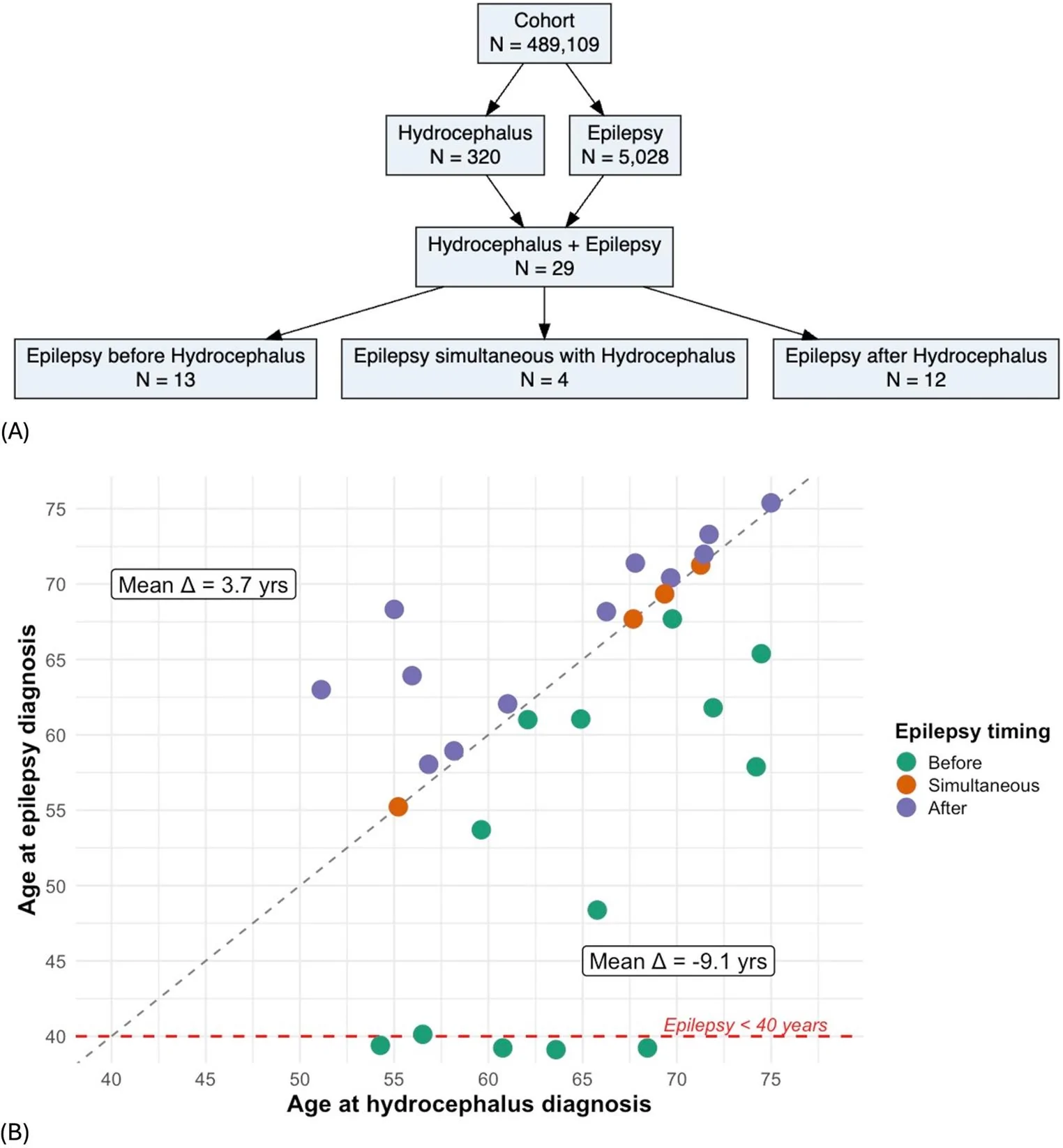
Temporal relationship between hydrocephalus and epilepsy diagnoses. Panel A shows the classification of participants with both hydrocephalus and epilepsy (n=29) according to the relative timing of diagnoses: epilepsy preceding hydrocephalus (n=13), diagnosed in the same year (n=4), or following hydrocephalus (n=12). Panel B displays a scatter plot of age at hydrocephalus diagnosis versus age at epilepsy diagnosis for these participants. Points are color-coded by timing category (before, simultaneous, after). The diagonal dashed line represents equal ages at the time of diagnosis. The horizontal red dashed line marks the exclusion threshold for epilepsy diagnosed at age ≤ 40 years, reflecting the study’s focus on late-onset cases. Mean age differences (Δ) between conditions are indicated for each group and cases with epilepsy onset before 40 years of age were not included

Logistic regression analysis revealed a statistically significant association between adult-onset hydrocephalus and epilepsy, independent of age and sex (OR = 9.6; 95% CI: 6.4–13.8; *p* < 0.001). (Fig. 2) illustrates the temporal relationship between the diagnoses of hydrocephalus and epilepsy. Among the 29 participants with both hydrocephalus and epilepsy, epilepsy preceded hydrocephalus in 13/29 (44.8%), co-occurred in 4/29 (13.8%), and was diagnosed after hydrocephalus in 12/29 (41.4%). The mean interval between diagnoses was 9.1 years when hydrocephalus followed epilepsy and 3.7 years when epilepsy occurred after hydrocephalus. 

Subsequently, we evaluated the risk of developing epilepsy after a diagnosis of hydrocephalus using a sampled cohort design. Cox proportional hazards models demonstrated a substantially increased risk of incident epilepsy in people with adult-onset hydrocephalus, with HRs ranging from 18.39 (95% CI: 10.05–33.65, *p* < 0.001) to 22.59 (95% CI: 12.22–41.74, *p* < 0.001) in the unadjusted model.

**Fig. 3:**
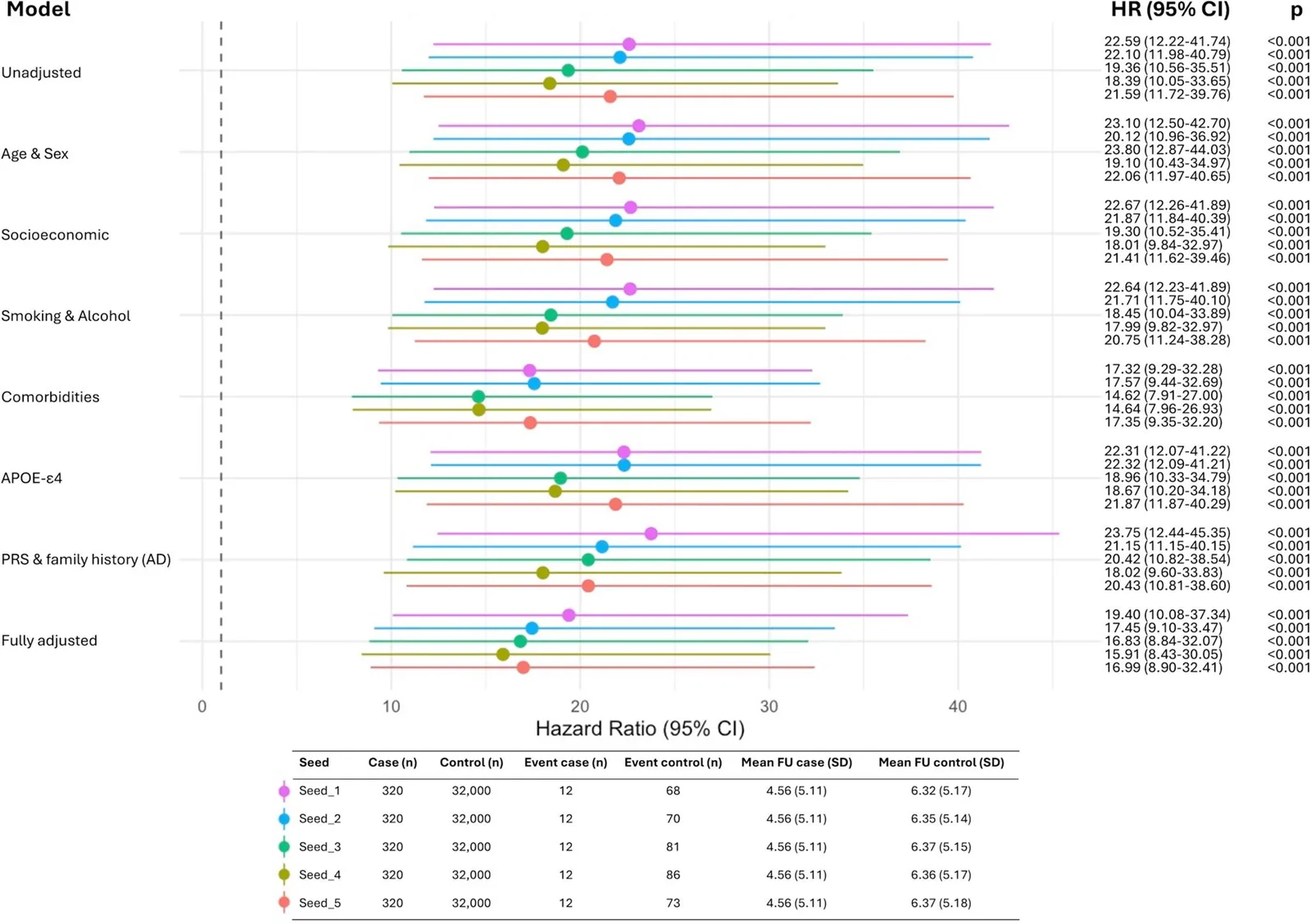
Cox proportional hazards models across five matched datasets (seeds 1-5).This forest plot shows hazard ratios (HRs) with 95% confidence intervals for the association between hydrocephalus and subsequent epilepsy, estimated across five independently matched datasets (Seeds 1-5). For each seed, Cox proportional hazards models were progressively adjusted, beginning with a base model, followed by sequential adjustment for age and sex, socioeconomic status (Townsend deprivation index) and ethnicity, lifestyle factors (smoking and alcohol consumption), clinical comorbidities (diabetes and hypertension), and genetic variables including APOE ε4 carrier status, polygenic risk score for Alzheimer’s disease, and family history of dementia

The inclusion of cardio-vascular and metabolic comorbidities slightly attenuated the association, but it remained largely significant, and the fully adjusted model, including several covariates, showed HRs ranging from 15.91 (95% CI: 8.43–30.05, *p* < 0.001) to 19.40 (95% CI: 10.08–37.34, *p* < 0.001) across seeds, as shown in (Fig. 3). The results remained consistent across five different matched datasets (seeds), showing no significant differences, supporting the robustness of the findings. The proportional hazards assumption was tested using Schoenfeld residuals for all models and seeds, with no significant violations detected (global p-values > 0.05), confirming the validity of the Cox models over time. 

Sensitivity analyses excluding participants with a clinically diagnosed Alzheimer’s disease and other neurodegenerative disorders (Supplementary Table 3) or after excluding potential seizure-mimicking conditions (Supplementary Table 4) yielded comparable HRs.

## Discussion

In this large, population-based cohort, logistic regression results demonstrated a strong association between adult-onset hydrocephalus and epilepsy. In addition, survival analyses using a sampled cohort design showed a markedly increased rate of incident epilepsy following the diagnosis of hydrocephalus, suggesting a temporal association between the two conditions.

Epilepsy in older age is commonly associated with cerebrovascular disease and neurodegeneration, which represent the most frequently identified causes of LOE [29, 30]. However, many cases are unexplained after standard evaluation [30], and evidence regarding epilepsy in potentially reversible neurological conditions, such as iNPH, remains lacking. In this study, individuals with adult-onset hydrocephalus had an increased risk of epilepsy, suggesting that hydrocephalus may also contribute to the broad spectrum of LOE. Notably, none of the subjects with both hydrocephalus and epilepsy in the study cohort had undergone shunt placement, supporting that the observed association with epilepsy is unlikely to be explained by shunt-related complications.

Several plausible pathways might explain this association. One potential mechanism involves cerebrovascular vulnerability: vascular risk factors, such as hypertension and diabetes, and small-vessel disease are frequently observed in both idiopathic hydrocephalus and LOE [31, 32]. In our analysis, however, adjusting for diabetes and hypertension slightly reduced the HRs, but the risk remained significantly high, indicating that metabolic and cardiovascular comorbidities may contribute but do not fully explain the increased epilepsy risk in hydrocephalus.

Another proposed explanation relates to structural and physiological consequences of ventricular enlargement [33, 34]. Altered cerebrospinal fluid dynamics and the resulting distortion of surrounding brain tissue may influence cortical excitability [35], particularly in temporal and limbic regions, frequently involved in focal epilepsy [36, 37]. Although plausible, this interpretation remains speculative and requires further investigation through targeted studies aiming to clarify the pathophysiological mechanisms underlying the observed association.

Our results could be clinically relevant for several reasons. In routine clinical appointments, subtle epileptic seizures, such as those presenting only with mild focal impaired awareness or brief behavioural arrest, may easily go unrecognized in people with hydrocephalus, particularly when attention is focused on more prominent features such as cognitive impairment, gait disturbance, or movement disorders. In this cohort study, approximately 9% of individuals with adult-onset hydrocephalus had a diagnosis of epilepsy (29 out of 320 cases), which is substantially higher than the prevalence of epilepsy in the general aging population [30, 38]. This finding suggests that neurologists, especially those not specialised in epilepsy, should actively look for seizures in people with hydrocephalus.

Another relevant clinical question concerns the response to shunt treatment. While postoperative epilepsy has been reported following shunt placement [19], the procedure, given the potentially reversible nature of idiopathic hydrocephalus, might conversely improve pre-existing epilepsy in some individuals or prevent future seizure appearance. This intriguing possibility has not yet been examined and may deserve further investigation.

The current study primarily aimed to to investigate whether adult-onset hydrocephalus was associated with an increased occurrence of subsequent late-onset epilepsy. In our cohort, however, we also observed a number of subjects with epilepsy occurring simultaneously or before NPH diagnosis, whole interpretation needs caution. NPH is often characterized by an indolent disease course (differently from seizures) and is associated with significant diagnostic delay; moreover, as observed in other dementia, epileptic seizures may be the first manifestation of other diseases, whose diagnosis is refined later. Therefore, in cases where seizures preceded the diagnosis of hydrocephalus by only a few years, we cannot exclude that the hydrocephalic process was already underway at a subclinical or minimally symptomatic stage.

The current study has several strengths, including the use of a very large cohort, stringent criteria to minimize secondary or misclassified cases, and replication of results across five independently seeded matched datasets. Moreover, we conducted a comprehensive sensitivity analysis including demographic, socioeconomic, lifestyle, vascular, genetic, and family history covariates, which supported the stability of the observed association. Importantly, the results are consistent even after excluding individuals with a clinical diagnosis of neurodegenerative disorders and dementia, including Alzheimer’s disease, a condition in which epilepsy is increasingly recognized as part of the clinical spectrum [39, 40], and after excluding subjects with seizure mimics, strengthening our findings. Still, future studies using biologically defined Alzheimer’s disease or cases with post-mortem AD pathological diagnosis are needed to confirm our results. Finally, another strength is the multidisciplinary approach, which brings together experts in both epilepsy and hydrocephalus. We strongly support continuous interplay between different neurological specialists, which is often lacking in dedicated clinics. Such collaboration can reveal potential disease associations that might otherwise go unnoticed.

Several limitations should also be acknowledged. First, the diagnoses of hydrocephus and epilepsy were derived from health record ICD-10 codes and may therefore be subject to misclassification; moreover, comorbidity of neurodegenerative diseases in patients with hydrocephalus may also occurr. Unfortunately, the absence of detailed cerebrospinal fluid biomarkers, amyloid PET imaging, comprehensive neuropsychological assessment and EEG evaluation represents an intrinsic limitation of the UK Biobank dataset. However, the association between adult-onset hydrocephalus and incident epilepsy remained highly significant after excluding all subjects with a diagnosis of AD and a broader spectrum of parkinsonism or dementia syndromes which may also mimic or coexist with hydrocephalus, and subjects with conditions potentially mimicking epileptic seizures, suggesting that misdiagnosis or comorbidities are unlikely to drive our findings. Overall, the current study provides insights on a new observation which needs to be further investigated in future tailored prospective studies.

Second, we acknowledge the lack of epilepsy phenotyping since most cases of interest were coded as unspecified epilepsy with no information on the seizure type. Third, the potential side effects of antiseizure medications on cognition may represent another relevant source of bias to be acknowledged. However, most cases were on monotherapy and taking anti-seizure medications (carbamazepine or valproid acid) not significantly impacting cognition [41]. Finally, this is a retrospective analysis of cross-sectional UK Biobank data, which limits the ability to infer a causal relationship between hydrocephalus and epilepsy. Nonetheless, the consistency of results across analytic strategies supports the robustness of the observed association and justifies prospective studies to corroborate and further expand our outcomes.

In summary, our findings indicate that adult-onset hydrocephalus is associated with an increased risk of epilepsy, independent of surgical treatment and not entirely explained by vascular or neurodegenerative comorbidities. These observations highlight the need for greater diagnostic vigilance in this population and support further investigation using advanced imaging and neurophysiological approaches to clarify the underlying mechanisms and guide more personalised therapeutic strategies. 

## Supplementary Information

Below is the link to the electronic supplementary material.

Supplementary Material 1

## Data Availability

The UK Biobank is an open-access resource available at https://www.ukbiobank.ac.uk/researchers/. Data can be obtained from the UK Biobank by submitting a data request proposal. The data supporting this study’s findings were used under license for the current study (Application #147093) and are not publicly available.
