# Supplementary material for: Risk of epilepsy in people with adult-onset hydrocephalus: insights from the UK Biobank
Source: Neurol Sci. 2026 Jul 16;47(8):635. doi: 10.1007/s10072-026-09227-6 (PMC13372835; doi:10.1007/s10072-026-09227-6)
Supplement: Supplementary file 1 — Supplementary Material 1 [file 10072_2026_9227_MOESM1_ESM.docx]

**Supplementary Methods**

*Control selection and follow-up strategy*

To evaluate the association between hydrocephalus and epilepsy, controls were defined as all individuals without a recorded diagnosis of hydrocephalus. To ensure comparability in the definition of time-at-risk between cases and controls, we generated a pseudo-index date for each control using the following procedure:

- we first took the ages at diagnosis observed among individuals with hydrocephalus and used this distribution to generate plausible diagnosis ages for controls. Each control was then assigned an age randomly sampled from this distribution;
- this sampled age was added to the control’s date of birth to obtain a corresponding calendar date, which served as the pseudo-index date;
- a fixed random seed (42) was used to ensure reproducibility;
- negative or implausible intervals (e.g. pseudo-index dates falling after the censoring date or yielding invalid follow-up times) were excluded.

This procedure was applied consistently across both analytic approaches. In logistic regression models, the pseudo-index date was used solely to harmonize the age distribution of cases and controls, as the outcome was defined as the presence of epilepsy regardless of temporal sequence. In Cox proportional hazards models, the index date marked the start of follow-up, defined as the actual hydrocephalus diagnosis date for cases and the pseudo-index date for controls.

Time at risk was calculated from the index (or pseudo-index) date until the earliest of: epilepsy diagnosis, death, or administrative censoring. Only epilepsy diagnoses occurring after the index/pseudo-index date were considered events in the survival analyses. Censoring dates were defined according to the last available follow-up in each country: 31 October 2022 for England, 31 August 2022 for Scotland, and 31 May 2022 for Wales. To assess the stability of the findings and quantify sampling variability, control selection was repeated using five independent random seeds, generating parallel analytic datasets. Robustness of hazard ratio estimates was evaluated both within datasets (across adjustment models) and between datasets. Cross-dataset variation was tested using the Kruskal-Wallis test, followed, where applicable, by Dunn’s post-hoc comparisons with Bonferroni correction.

*Statistical models*

The association between hydrocephalus and epilepsy was first estimated using logistic regression, with model specification:

logit(P(Epilepsy_i=1)) = β0 + β1 * Hydrocephalus_i + β2 * Age_i + β3 * Sex_i

where P(Epilepsy_i=1) denotes the probability of epilepsy for participant i. Hydrocephalus was included as a binary exposure (case vs. control), while age at index (age at diagnosis for cases or at pseudo-index date for controls) and sex were included as covariates. Odds ratios (ORs) with 95% confidence intervals (CIs) were obtained by exponentiating model coefficients.

For longitudinal analyses, Cox proportional hazards models were specified as:

h_i(t) = h0(t) * exp(β1 * Hydrocephalus_i + β2 * Age_i + β3 * Sex_i + … + βp * X_pi)

where h_i(t) is the hazard of epilepsy at time t for individual i, h0(t) is the baseline hazard, and X_pi represents additional covariates. Hazard ratios (HRs) with 95% CIs were derived from exponentiated coefficients, and proportional hazards assumptions were tested using Schoenfeld residuals.

*Cox Proportional Hazards Models*

We estimated a sequence of Cox proportional hazards models with progressive adjustment for potential confounders. The models were defined as follows:

- Model 1 (Base): hydrocephalus status only.
- Model 2 (Age & Sex): adjusted for age at index and sex.
- Model 3 (Socioeconomic): adjusted for Townsend deprivation index and ethnicity.
- Model 4 (Lifestyle): adjusted for smoking status and alcohol consumption.
- Model 5 (Vascular comorbidities): adjusted for diabetes and hypertension.
- Model 6 (APOE): adjusted for APOE ε4 carrier status.
- Model 7 (Genetics & Family history): adjusted for polygenic risk score (PRS) for Alzheimer’s disease and family history of dementia in first-degree relatives.
- Model 8 (Fully adjusted): included all covariates simultaneously.

Most covariates (sex, ethnicity, smoking, alcohol consumption, diabetes, hypertension, APOE ε4 carrier status, and family history of dementia) were naturally dichotomous or were recoded into binary indicators, reflecting the presence or absence of a given characteristic. In contrast, the Townsend deprivation index and the polygenic risk score (PRS) for Alzheimer’s disease are continuous measures. Age at index was also retained as a continuous variable (years).

Adjustment for Alzheimer-related markers (APOE ε4, PRS, and family history) was motivated by the need to isolate the risk of epilepsy specifically attributable to hydrocephalus. Epilepsy is more common in individuals with Alzheimer’s disease or a genetic predisposition to dementia, and hydrocephalus itself often co-occurs with cognitive decline. Without accounting for this overlap, any excess risk of epilepsy observed in patients with hydrocephalus could partly reflect underlying neurodegenerative processes. By adjusting for Alzheimer-related genetic and familial factors, we aimed to reduce this potential confounding and provide a clearer estimate of the independent association between hydrocephalus and subsequent epilepsy.

| **ICD-10 code** | **Diagnosis** | **Rationale** |
| --- | --- | --- |
| *Secondary causes* |  |  |
| C71, D33.0, D33.1, D33.2, R90.0 | Malignant and benign neoplasm of brain | May cause secondary hydrocephalus and/or structural epilepsy |
| G80 | Infantile cerebral palsy | Major congenital condition strongly associated with hydrocephalus/epilepsy |
| Q00-07 | Congenital malformations of the nervous system | Includes spina bifida, anencephaly; relevant for secondary hydrocephalus. |
| Q85 | Phakomatoses | Genetic disorders predisposing to brain lesions and epilepsy |
| Q03, G91.1, G91.3, G94.0, G94.1 | Secondary and other hydrocephalus; | Non-idiopathic forms of hydrocephalus |
| F70-79 | Intellectual disability | Strongly associated with early structural brain abnormalities and/or monogenic epilepsy |
| S02, S06 (1-9), S07, S09 (7-8) | Skull and intracranial injuries | Major head trauma that may cause secondary hydrocephalus and/or structural epilepsy |
| G00-03 | Bacterial and non-bacterial meningitis* | May cause secondary hydrocephalus and/or structural epilepsy |
| I60-I64, I67, I69 | Cerebrovascular diseases (haemorrhage, infarction)* | May cause secondary hydrocephalus and/or structural epilepsy |
| *Age-based exclusion* | |  |
| G40, G41 | Epilepsy and status epilepticus | Excluded if onset age ≤2 years (focus on late-onset cases) |
| G91 | Hydrocephalus | Excluded if diagnosed at age ≤40 years (to focus on adult/late-onset cases) |

**Supplementary Table 1.** ICD‑10 codes excluded from the analysis and rationale.

This table lists the ICD‑10 codes excluded from the study cohort, grouped by the reason for exclusion. Secondary causes include conditions with a well‑established potential to directly cause hydrocephalus and/or epilepsy (e.g., neoplasms, congenital malformations, severe head trauma, etc).

*For meningitis, encephalitis and cerebrovascular disease the diagnosis date was available; this allowed to exclude only cases where these conditions occurred prior to or within 1 month from the hydrocephalus and/or epilepsy diagnoses (the latest, if both present); indeed, the occurrence of such conditions (i.e. stroke) after hydrocephalus and/or epilepsy diagnoses was not deemed relevant to study the association between hydrocephalus and epilepsy, which was the aim of the current study, thus these patients were not excluded. This allowed for avoiding significant sample size reduction while using a rigorous patient selection procedure to minimize the effect of potential confounding factors. Age‑based exclusions were applied to minimize inclusion of conditions less likely to represent idiopathic normal pressure hydrocephalus (hydrocephalus onset ≤ 40 years) or late-onset epilepsy (epilepsy onset ≤ 2 years, where monogenic aetiology is likely).

| **Variable** | **Variable description** | **UK Biobank Field ID(s)** |
| --- | --- | --- |
| *Age at baseline visit* | Age in years at the initial UK Biobank assessment visit. | 21022 |
| *Age at hydrocephalus diagnosis* | Age at first recorded ICD-10 diagnosis of hydrocephalus (G91) from linked hospital inpatient or primary care records. Derived from event  date and date of birth. | 131110, 34, 52 |
| *Age at epilepsy diagnosis* | Age at first recorded ICD-10 diagnosis of epilepsy (G40) from linked hospital inpatient or primary care records. Derived from event date  and date of birth. | 131048, 34, 52 |
| *Townsend Deprivation Index* | aAcomposite measure of socioeconomic deprivation based on unemployment, non-home ownership, household overcrowding, and lack of car access, with greater values indicating more deprivation | 22189 |
| *Ethnicity* | Self-reported ethnic background. | 21000 |
| *Current smokers* | Participants who reported being current smokers at the baseline visit. | 20116 |
| *Current alcohol* | Participants who reported current alcohol consumption at baseline. | 20117 |
| *Diabetes* | Participants with an ICD-10 diagnosis of diabetes mellitus. | 41270 (ICD-10 codes: E10, E11, E12, E13, E14, O24.0, O24.1, O24.3) |
| *Hypertension* | Participants with an ICD-10 diagnosis of hypertension. | 41270 (ICD-10 codes: I10, I11, I12, I13, I15) |
| *ApoE-ε4 carrier* | Participants carrying at least one *APOE ε4* allele (rs429358 or rs7412) determined from imputed genotype data. | 22000 |
| *Family history of AD/dementia* | Participants reporting that at least one of first-degree relatives (parents, siblings) had Alzheimer’s disease or other dementia. | 20107, 20110, 20111 |
| *PRS for AD* | Polygenic risk score for Alzheimer’s disease, calculated from imputed genome-wide data using the most recent GWAS summary statistics. | 26206 |

**Supplementary Table 2.** Variables included in the study and their corresponding UK Biobank Field IDs.

Ages at hydrocephalus and epilepsy diagnosis were derived from the date of first ICD-10 code registration (fields 131110 and 131048, respectively) and participants’ date of birth (fields 34 and 52). The Townsend Deprivation Index represents area-level socioeconomic status, with higher scores indicating greater deprivation. Family history for AD/dementia was determined based on participant-reported information on illnesses diagnosed in first-degree relatives, specifically in the father (Field ID: 20107), mother (Field ID: 20110), and siblings (Field ID: 20111). A family history of AD was assigned when this disease was reported in at least one of these relatives. The polygenic risk score for Alzheimer’s disease was computed using genome-wide association study summary statistics from the most recent large-scale meta-analysis. *Abbreviations: AD = Alzheimer’s disease; ApoE-ε4= apolipoprotein E epsilon-4 allele; ICD-10 = International Classification of Diseases, 10th Revision; PRS = polygenic risk score; GWAS = genome-wide association study.*

| **Model** | |  | **HR** | **CI 95%** | **p value** |
| --- | --- | --- | --- | --- | --- |
| Unadjusted | | Seed 1 | 17.53 | 7.46-41.21 | <0.001 |
|  |  | Seed 2 | 15.79 | 6.75-36.95 | <0.001 |
|  |  | Seed 3 | 18.63 | 7.91-43.88 | <0.001 |
|  |  | Seed 4 | 14.72 | 6.33-34.24 | <0.001 |
|  |  | Seed 5 | 14.76 | 6.34-34.35 | <0.001 |
| Age & Sex | | Seed 1 | 17.99 | 7.65-42.30 | <0.001 |
|  |  | Seed 2 | 16.31 | 6.97-38.18 | <0.001 |
|  |  | Seed 3 | 19.49 | 8.26-45.97 | <0.001 |
|  |  | Seed 4 | 15.36 | 6.60-35.75 | <0.001 |
|  |  | Seed 5 | 15.40 | 6.61-35.85 | <0.001 |
| Socioeconomic | | Seed 1 | 17.43 | 7.41-40.97 | <0.001 |
|  |  | Seed 2 | 15.79 | 6.75-36.97 | <0.001 |
|  |  | Seed 3 | 18.15 | 7.70-42.77 | <0.001 |
|  |  | Seed 4 | 14.43 | 6.20-33.58 | <0.001 |
|  |  | Seed 5 | 15.01 | 6.44-34.95 | <0.001 |
| Smoking & Alcohol | | Seed 1 | 17.46 | 7.42-41.09 | <0.001 |
|  |  | Seed 2 | 15.72 | 6.71-36.81 | <0.001 |
|  |  | Seed 3 | 18.63 | 7.90-43.96 | <0.001 |
|  |  | Seed 4 | 13.94 | 5.98-32.49 | <0.001 |
|  |  | Seed 5 | 14.38 | 6.17-33.51 | <0.001 |
| Comorbidities | | Seed 1 | 13.53 | 5.71-32.06 | <0.001 |
|  |  | Seed 2 | 14.15 | 5.99-33.47 | <0.001 |
|  |  | Seed 3 | 15.28 | 6.44-36.24 | <0.001 |
|  |  | Seed 4 | 11.23 | 4.80-26.29 | <0.001 |
|  |  | Seed 5 | 11.71 | 5.01-27.37 | <0.001 |
| APOE-ε4 | | Seed 1 | 17.31 | 7.36-40.67 | <0.001 |
|  |  | Seed 2 | 16.00 | 6.83-37.46 | <0.001 |
|  |  | Seed 3 | 18.63 | 7.91-43.89 | <0.001 |
|  |  | Seed 4 | 14.74 | 6.33-34.29 | <0.001 |
|  |  | Seed 5 | 14.89 | 6.40-34.66 | <0.001 |
| PRS & family history (AD) | | Seed 1 | 19.78 | 8.32-47.00 | <0.001 |
|  |  | Seed 2 | 17.10 | 7.28-40.19 | <0.001 |
|  |  | Seed 3 | 21.30 | 9.00-50.42 | <0.001 |
|  |  | Seed 4 | 15.61 | 6.69-36.44 | <0.001 |
|  |  | Seed 5 | 16.38 | 7.01-38.27 | <0.001 |
| Fully adjusted | Seed 1 | 15.68 | 6.54-37.63 | <0.001 |  |
|  | Seed 2 | 16.70 | 7.03-39.66 | <0.001 |  |
|  | Seed 3 | 18.95 | 7.92-45.36 | <0.001 |  |
|  | Seed 4 | 13.00 | 5.52-30.62 | <0.001 |  |
|  | Seed 5 | 14.85 | 6.31-34.94 | <0.001 |  |

**Supplementary Table 3.** Sensitivity analysis: association between adult-onset hydrocephalus and incident epilepsy after excluding participants with Alzheimer’s disease and other neurodegenerative disorders and dementias.

Five matched datasets (Seeds 1–5) were generated after removal of all participants with clinically diagnosed neurodegenerative disorders and dementias, including Alzheimer’s disease (ICD-10: G30, F00), vascular dementia (F01), dementia in other diseases (F02), unspecified dementia (F03), Parkinson’s disease (G20), secondary parkinsonism (G21), other degenerative diseases of the basal ganglia, including atypical parkinsonian syndromes (G23), and other degenerative diseases of the nervous system, including frontotemporal dementia-related disorders (G31). Cox proportional hazards models corresponding to those used in the main analysis were applied. Hazard ratios and 95% confidence intervals are shown for each seed and adjustment level. Results remained consistent with the primary analysis, supporting the robustness of the association. *Abbreviations: PRS = polygenic risk score; AD = Alzheimer’s disease; HR = hazard ratio; CI = confidence interval.*

| **Model** |  | **HR** | **CI 95%** | **p value** |
| --- | --- | --- | --- | --- |
| Unadjusted | Seed 1 | 26.23 | 13.25-51.92 | <0.001 |
|  | Seed 2 | 24.44 | 12.40-48.15 | <0.001 |
|  | Seed 3 | 22.43 | 11.44-43.97 | <0.001 |
|  | Seed 4 | 20.05 | 10.30-39.06 | <0.001 |
|  | Seed 5 | 25.89 | 13.11-51.13 | <0.001 |
| Age & Sex | Seed 1 | 26.49 | 13.38-52.45 | <0.001 |
|  | Seed 2 | 24.68 | 12.52-48.65 | <0.001 |
|  | Seed 3 | 22.83 | 11.64-44.79 | <0.001 |
|  | Seed 4 | 20.55 | 10.55-40.05 | <0.001 |
|  | Seed 5 | 26.04 | 13.18-51.45 | <0.001 |

**Supplementary Table 4.** Sensitivity analysis: association between adult-onset hydrocephalus and incident epilepsy after excluding participants with seizure-mimicking conditions.

To reduce the possibility of diagnostic misclassification, participants with epilepsy diagnoses and concomitant ICD-10 codes corresponding to potential seizure-mimicking conditions, including syncope and collapse (R55), transient alteration of awareness (R40.4), dizziness and giddiness (R42), transient ischaemic attacks and related syndromes (G45), and heatstroke/sunstroke (T67), were excluded. Five matched datasets (Seeds 1–5) were generated and analysed using Cox proportional hazards models. Hazard ratios and 95% confidence intervals are reported for unadjusted and age- and sex- adjusted models. Results remained consistent with the primary analysis, supporting the robustness of the association. *Abbreviations: HR = hazard ratio; CI = confidence interval.*
